# Shouldering the challenge of deciphering avian palate evolution

**DOI:** 10.1073/pnas.2514111123

**Published:** 2026-01-02

**Authors:** Juan Benito, Pei-Chen Kuo, Christopher R. Torres, Guillermo Navalón, Olivia Plateau, Alexander D. Clark, Elizabeth M. Steell, Daniel J. Field

**Affiliations:** ^a^Department of Earth Sciences, University of Cambridge, Cambridge CB2 3EQ, United Kingdom; ^b^Negaunee Integrative Research Center, Field Museum of Natural History, Chicago, IL 60605; ^c^Department of Biological Sciences, University of the Pacific, Stockton, CA 95211; ^d^Department of Life Sciences, Universidad de Alcalá, Alcalá de Henares, Madrid 28805, Spain; ^e^The Institute of Ecology and Evolution, University of Bern, Bern 63012, Switzerland; ^f^Naturhistorisches Museum Bern, Bern 3005, Switzerland; ^g^Museum of Zoology, University of Cambridge, Cambridge CB2 3EJ, United Kingdom

Wilken et al. (1) investigate the evolution of avian palatal kinesis using comparative morphology and biomechanical modeling. While the study’s topic and approach are timely, its conclusions are marred by inadequate taxon sampling and morphological misinterpretations.

**Fig. 1.**
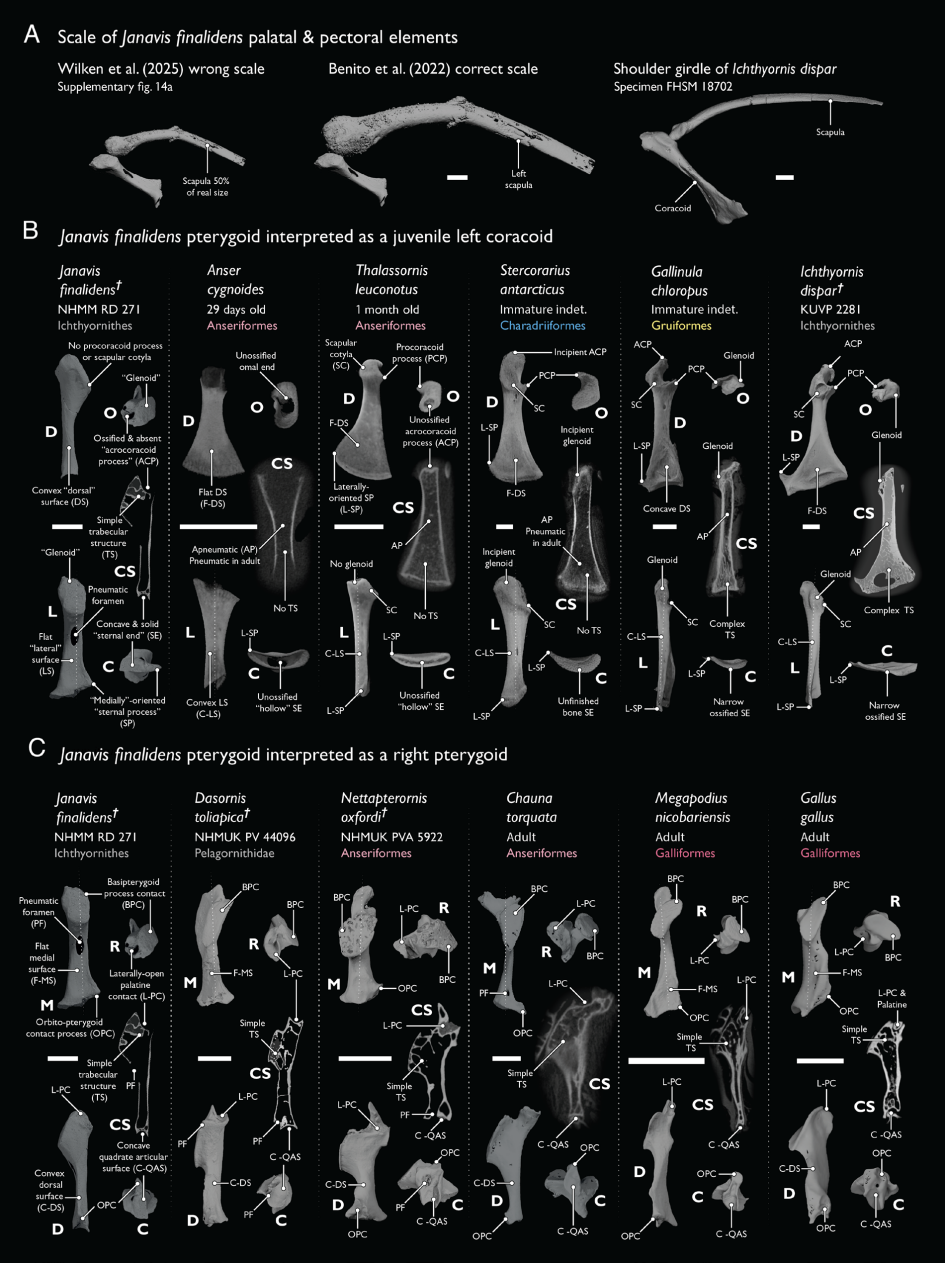
The pterygoid of *Janavis finalidens* is not a juvenile coracoid. (*A*) The scapula of *Janavis* was downscaled to 50% of its real size by (1) (see their *SI Appendix*, Figure S14). Correctly scaled, the scapula cannot form a plausible scapulocoracoid with the pterygoid (Figure 1*A* and *SI Appendix*, Figure S2 B–D in ref. 2). (*B*) If interpreted as an immature bird coracoid as advocated by Wilken et al. (1), it would be unique among all extant and fossil birds in numerous aspects of its morphology, the order specific structures appear (i.e., glenoid before scapular cotyle and procoracoid), and its high degree of ossification. (*C*) If correctly interpreted as a right pterygoid, all features of the element are found in fossil and extant neognathous birds. Bold letters indicate anatomical views: caudal (C), dorsal (D), lateral (L), medial (M), and rostral (R). Cross-sectional (CS) planes are indicated with dotted lines over each element. (Scale bars equal 5 mm.)

Reinterpreting the *Janavis* pterygoid (2) as a juvenile bird coracoid (1) is contradicted by clear morphological and developmental evidence (Fig. 1). The concave, ossified “sternal articulation” of the would-be coracoid (1) is unossified in juvenile bird coracoids, yet resembles the quadrate articulation of the pterygoid in several extant taxa (Fig. 1*B*). Similarly, *contra* (1), the basipterygoid articulation in *Janavis* closely resembles that of several extant neognathous birds in its form and position (Fig. 1*C*).

The authors identify a supposed glenoid and acrocoracoid on the purported coracoid (1), yet in developing bird coracoids these structures only arise after the scapular cotyle, which is absent in the fossil. Importantly, the positions of these structures do not shift relative to one another throughout extant bird ontogeny, which would be necessary for the element to come to resemble a coracoid (Fig. 1*B*). Since ichthyornithine postcranial morphology and growth generally fall within the range of variation of extant birds (3), there is no reason to think these would be any different in *Janavis*. Crucially, the authors of Wilken et al. (1) must have reduced the size of the *Janavis* scapula by ~50% to “match” it with the putative coracoid (see their *SI Appendix*, Figure S14*A*; our Fig. 1*A*), yet this is unmentioned.

**Fig. 2.**
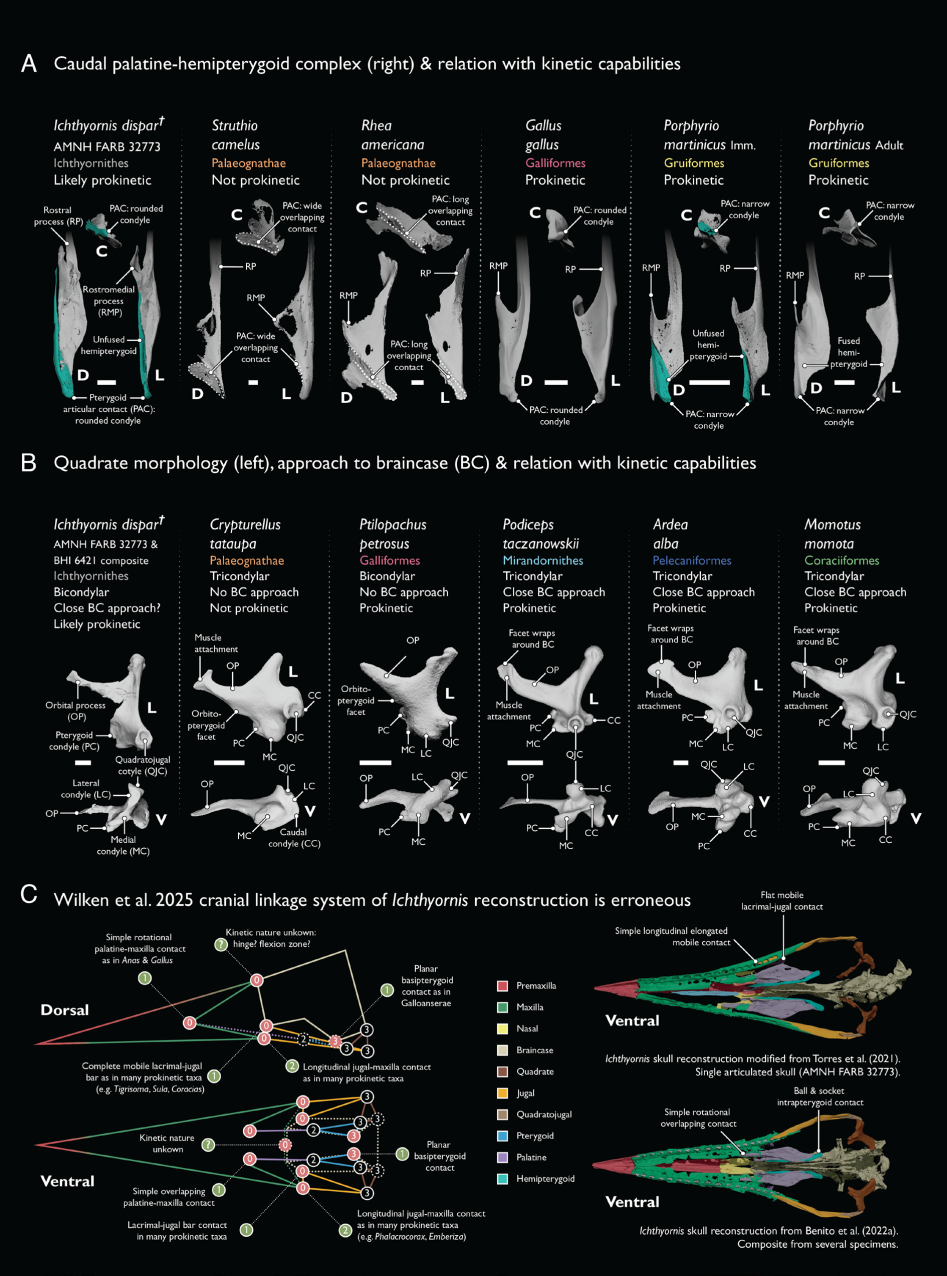
Morphology of the ichthyornithine suspensorial system suggests prokinetic capabilities. (*A*) *Ichthyornis* exhibited a mobile intrapterygoid (hemipterygoid-pterygoid) articulation, like in immature (imm.) neognaths; its condylar nature resembles prokinetic Galliformes and Neoaves, contrasting with the long or wide overlapping contacts of paleognaths. Hemipterygoids highlighted in teal. Long rostral processes of the palatine obscured for clarity. (*B*) The quadrates of *Ichthyornis* and *Hesperornis* [bicondylar, with long orbital processes interpreted as contacting the braincase (1)] would not preclude palatal kinesis. Both features are present in a wide range of prokinetic neognath taxa unsampled by Wilken et al. (1) (see 10.5281/zenodo.15685484). (*C*) The ichthyornithine linkage models proposed by Wilken et al. (1) enforce immobility at contacts between bones (e.g., the jugal and the lacrimal) which do not preclude prokinesis in extant birds. Corrected linkage values shown in green. Bold letters indicate anatomical views as in Fig. 1. (Scale bars equal 2.5 mm.) Segmented *Struthio* palatine courtesy of Annabel Hunt.

The above issues notwithstanding, the ichthyornithine hemipterygoid alone—with an articular condyle like those of prokinetic neognaths—suggests a mobile intrapterygoid joint (Fig. 2*A*) (4). Wilken et al. seemingly agree [“…*Ichthyornis...* possesses a segmented, potentially propulsive pterygoid...” (1)]; yet, they interpret palatal kinesis as a neognath autapomorphy by asserting that stem bird musculature was incompatible with palate mobility. This rests upon misinterpretations of the functional correlates of quadrate morphology. First, elongate orbital processes in *Ichthyornis* and *Hesperornis* are declared to impose constraints on kinesis due to articulation with the neurocranium (1). However, many neognaths exhibit equivalently long (or longer) orbital processes closely approaching the neurocranium without precluding prokinesis [Fig. 2*B*; (5); 10.5281/zenodo.15685484] Second, *contra* (1), the bicondylar nature of the *Ichthyornis* quadrate would not have precluded coupled palatal kinesis, as evidenced by bicondylar Anseriformes (Fig. 2*B*) (6). Further morpho-functional misconceptions underpin problematic cranial linkage models in Wilken et al. (1) (Fig. 2*C*), yet the authors’ own ancestral reconstructions of cranial linkages and protractor force seemingly support palatal mobility as plesiomorphic for all crown birds (their figures 3*A* and 4), rather than being apomorphic for Neognathae as they argue.

Contemporary research in avian macroevolution seeks to evaluate associations between brain and skull transformations (7–9); however, this link is weakly substantiated by Wilken et al. (1), as several taxa with palatal kinesis (e.g., Anseriformes) share plesiomorphically low degrees of encephalization with akinetic paleognaths and stem birds (10). The origin of palatal kinesis was a key milestone in avian evolutionary history—careful morphological interpretation will be crucial to clarify how this major transformation occurred and influenced avian evolutionary history.
